# Neuroinflammation in Lyme neuroborreliosis affects amyloid metabolism

**DOI:** 10.1186/1471-2377-10-51

**Published:** 2010-06-22

**Authors:** Niklas Mattsson, Daniel Bremell, Rolf Anckarsäter, Kaj Blennow, Henrik Anckarsäter, Henrik Zetterberg, Lars Hagberg

**Affiliations:** 1 Clinical Neurochemistry Laboratory, Institute of Neuroscience and Physiology, Department of Psychiatry and Neurochemistry, The Sahlgrenska Academy, University of Gothenburg, Mölndal, Sweden; 2 Department of Infectious Diseases, Sahlgrenska University Hospital, Gothenburg; 3 Department of Anaesthesiology and Intensive Care, Kungälv Hospital, Kungälv, Sweden; 4 Institute for Clinical Sciences, Malmö University Hospital, Lund University, Sweden

## Abstract

**Background:**

The metabolism of amyloid precursor protein (APP) and β-amyloid (Aβ) is widely studied in Alzheimer's disease, where Aβ deposition and plaque development are essential components of the pathogenesis. However, the physiological role of amyloid in the adult nervous system remains largely unknown. We have previously found altered cerebral amyloid metabolism in other neuroinflammatory conditions. To further elucidate this, we investigated amyloid metabolism in patients with Lyme neuroborreliosis (LNB).

**Methods:**

The first part of the study was a cross-sectional cohort study in 61 patients with acute facial palsy (19 with LNB and 42 with idiopathic facial paresis, Bell's palsy) and 22 healthy controls. CSF was analysed for the β-amyloid peptides Aβ38, Aβ40 and Aβ42, and the amyloid precursor protein (APP) isoforms α-sAPP and β-sAPP. CSF total-tau (T-tau), phosphorylated tau (P-tau) and neurofilament protein (NFL) were measured to monitor neural cell damage. The second part of the study was a prospective cohort-study in 26 LNB patients undergoing consecutive lumbar punctures before and after antibiotic treatment to study time-dependent dynamics of the biomarkers.

**Results:**

In the cross-sectional study, LNB patients had lower levels of CSF α-sAPP, β-sAPP and P-tau, and higher levels of CSF NFL than healthy controls and patients with Bell's palsy. In the prospective study, LNB patients had low levels of CSF α-sAPP, β-sAPP and P-tau at baseline, which all increased towards normal at follow-up.

**Conclusions:**

Amyloid metabolism is altered in LNB. CSF levels of α-sAPP, β-sAPP and P-tau are decreased in acute infection and increase after treatment. In combination with earlier findings in multiple sclerosis, cerebral SLE and HIV with cerebral engagement, this points to an influence of neuroinflammation on amyloid metabolism.

## Background

The trans-membranous protein amyloid precursor protein (APP) has been intensely studied in Alzheimer's disease (AD), since it is the source of β-amyloid (Aβ) peptides, recognized as key-components in AD pathophysiology [1]. Although ubiquitously expressed, the physiological role of APP in the adult organism remains largely unknown. APP may undergo non-amyloidogenic cleavage at the α-site, which inhibits formation of Aβ and releases an extracellular soluble α-sAPP fragment. Alternatively, APP is processed by combined cleavages by β-secretase and γ-secretase, releasing Aβ and β-sAPP. Aβ peptides vary in length due to variability in the γ-secretase cleavage site. Although CSF levels of α-sAPP and β-sAPP generally correlate tightly [2], it is not known how these pathways are orchestrated *in vivo*. CSF levels of α-sAPP and β-sAPP are reduced in MS and cerebral systemic lupus erythematosus SLE [3], and even lower levels are seen in HIV patients with cerebral engagement [4].

Lyme neuroborreliosis (LNB) is caused by a central nervous system (CNS) infection by the tick-borne spirochete *Borrelia burgdorferi*. LNB is often manifested by cranial nerve engagement, and common clinical findings are facial nerve palsy and radiculitic pain [5,6]. Common laboratory findings are increased albumin ratio, indicating impaired blood-brain barrier function, and CSF monocytosis. In this study, we investigated CSF markers of amyloid metabolism and neural cell damage in LNB, to elucidate the influence of neuroinflammation on amyloid metabolism.

The project contained two clinical studies. The first was a cross-sectional study in patients with acute facial palsy caused by either LNB or idiopathic Bell's palsy. The second was a longitudinal prospective cohort-study, where LNB patients were followed with successive lumbar punctures to investigate time-dependent biomarker dynamics.

## Methods

### Study participants

We enrolled different study participants for the cross-sectional study and the longitudinal study. Participants included LNB patients, Bell's palsy patients and controls. Diagnostic criteria for LNB were: I. Clinical symptoms consistent with neuroborreliosis and alternative explanations excluded; II Inflammatory CSF with mononuclear cell count >5 × 10^6^/l and erythrocytes <100 × 10^6^/L; III. One or more of the following: a) Intrathecal antibody production against *B burgdorferi sp*. b) Antibodies against *B burgdorferi sp*. in serum. c) Erythema migrans within three months; and IV. One or more of the following: a) CSF albumin >400 mg/L. b) Oligoclonal IgG and/or IgM-synthesis on CSF protein electrophoresis. c) IgG index (CSF/serum IgG ratio)/(CSF/serum albumin ratio) > .70. Bell's palsy (idiopathic facial palsy) was defined as acute, monosymptomatic, unilateral peripheral facial paresis of unknown etiology. Bell's palsy patients were included as non-infectious palsy controls.

In the cross-sectional part of the study we investigated 61 patients of whom 19 fulfilled criteria for LNB and 42 were classified as Bell's palsy. Twenty-two individuals for whom CSF analysis was done because of headache or vertigo, but infection and other diseases were excluded (CSF albumin and cell count were normal), served as controls.

The longitudinal study included 26 LNB patients with radiculitic pain and sensory disturbances. In addition, 3 patients had facial palsy, 3 had paraparesis, 1 had paresis of the accessorius nerve, and 1 had a trigeminus paresis. There was no overlap with the patients in the cross-sectional study. Ten patients without any neurological disorders, undergoing knee replacements, where CSF was drawn before surgery (Table 1) served as controls in the longitudinal study. These subjects are described in detail elsewhere [7]. All LNB patients were given oral treatment with doxycycline 200-400 mg daily for 10-14 days, which is the standard treatment in Sweden [8]. CSF was drawn before start of treatment and at follow-up. The median duration between the samplings was 45 days (range 33-61). All subjects gave informed consent to participate. The study was approved by the ethics committee of University of Gothenburg.

**Table 1:** Study participants and routine CSF analysis^a^

Group	N	M/F	Age years	Disease **duration**^**b**^	CSF monocytes **×10**^**6**^**/L**		CSF albumin ratio		CSF albumin (mg/l) mean (range)	
**Cross-sectional study**										

Controls	22	9/13	44 (25-67)	-	1 (1 - 34)		4.65 (2.7-10.5)		222 (83 - 411)	

LNB with facial palsy	19	11/8	42 (8 - 72)	21	136 (14 - 534)		16.3^c^ (3.8-49.9)		861^c^ (166 - 2850)	

Bell's palsy	42	18/24	36 (16-70)	5	2 (1 - 39)		4.7^d^ (2.3-11.5)		206^d^ (114 - 569)	

**Follow-up study**										

Controls	10	6/4	63 (51-70)	-	Missing data		6.4 (4.7-10.1)		302 (192 - 579)	

					**Baseline**	**Follow-up**	**Baseline**	**Follow-up**	**Baseline**	**Follow-up**

LNB	26	17/9	49 (12-74)	28	105 (14-590)	12 (2-21)	15^c^ (5.7-49.3)	6.1^e^ (4.7-13.6)	816^c^ (267-2180)	322^e^ (146-707)

CSF, cerebrospinal fluid; LNB, Lyme neuroborreliosis; ^a^data presented as median (range), if not stated otherwise; ^b^any neurologic symptom including radiculitic pain before study inclusion, presented as days (median); ^c^P < .001 vs controls; ^d^P < .001 vs LNB; ^e^P < .001 vs LNB baseline.

### Sampling

CSF samples were collected by lumbar puncture in the L3/L4 or L4/L5 interspace. Four mL of CSF was collected in a polypropylene tube and immediately transported to the local laboratory for centrifugation at 2.000 g at +4°C for 10 minutes. The supernatant was pipetted off, gently mixed to avoid possible gradient effects, and aliquoted in polypropylene tubes that were stored at -70°C pending biochemical analyses, without being thawed and re-frozen.

### Biochemical procedures

All biochemical analyses were performed at the Clinical Neurochemistry Laboratory in Mölndal, Sweden, by experienced laboratory technicians who were blinded to the clinical diagnoses and other clinical information.

### Markers of amyloid metabolism

CSF levels of Aβ38, Aβ40 and Aβ42 were measured using the MSD^® ^Human/Rodent (4G8) Abeta Triplex Assay as described by the manufacturer (Meso Scale Discovery, MSD^®^, Gaithersburg, MD, USA). This assay employs the 4G8 antibody to capture Aβ and C-terminal specific antibodies to specifically capture Aβ38, Aβ40 and Aβ42. All isoforms are detected by SULFO-TAG™-labeled anti-4G8 detection antibody. CSF concentrations of α-sAPP and β-sAPP were determined using the MSD^® ^sAPPα/sAPPβ Multiplex Assay as described by the manufacturer. This assay employs the 6E10 antibody to capture α-sAPP and a neoepitope-specific antibody to capture β-sAPP. Both isoforms are detected by SULFO-TAG™-labeled anti-APP antibody p2-1.

### Markers of neural cell damage

The axonal damage marker CSF T-tau was measured using a sandwich ELISA (INNOTEST^® ^hTAU-Ag, Innogenetics, Ghent, Belgium) specifically constructed to measure all tau isoforms irrespectively of phosphorylation status (T-tau), as previously described [9]. CSF concentrations of tau phosphorylated at threonine 181 (P-tau) was measured using a sandwich ELISA (INNOTEST^® ^PHOSPHO-TAU(181P), Innogenetics), as previously described [10]. CSF NFL, which is increased following damage to large myelinated axon, was analyzed using an ELISA, as previously described [11]. The detection limit for the NFL ELISA was 125 ng/L.

### Albumin

Quantitative determination of albumin in serum and CSF was performed using the Behring Nephelometer Analyser (Behringwerke AG, Marburg, Germany). The CSF/serum albumin ratio was calculated as: CSF albumin (mg/l)/serum-albumin (g/l).

### Statistical analyses

All statistical calculations were performed using SPSS 15.0 (SPSS Inc, Chicago, USA). As the distribution of quantitative measures was significantly skewed, statistical tests involving these variables were conducted using the non-parametric Kuskal-Wallis test for comparisons of multiple groups and the Mann-Whitney U test for pair-wise comparisons between groups. Quantitative variables are presented as median (range). The Spearman correlation coefficient was used for analyses of correlation between variable levels in different study groups. Statistical significance was determined at P < .05.

### Role of the funding source

The sponsors of the study had no role in study design, data collection, data analysis, data interpretation, or writing of the report. The corresponding author had full access to all the data in the study and had final responsibility for the decision to submit for publication.

## Results

In the cross-sectional study all groups were comparable in age. The only exception was that Bell's palsy patients were slightly younger than the controls (P = .023, Table 1). LNB patients had longer history of neurological symptoms before the time of lumbar puncture than Bell's palsy patients (Table 1).

In the longitudinal study, LNB patients were younger than the controls (P = .031, Table 1). The median duration of neurologic symptoms was 7 days longer than in the cross-sectional study (21 days compared with 28 days). At follow-up, all LNB patients had improved in their clinical symptoms and their inflammatory reactions had diminished, with decreased CSF monocytic cell counts (Table 1).

### Amyloid metabolism

**Figure 1:**
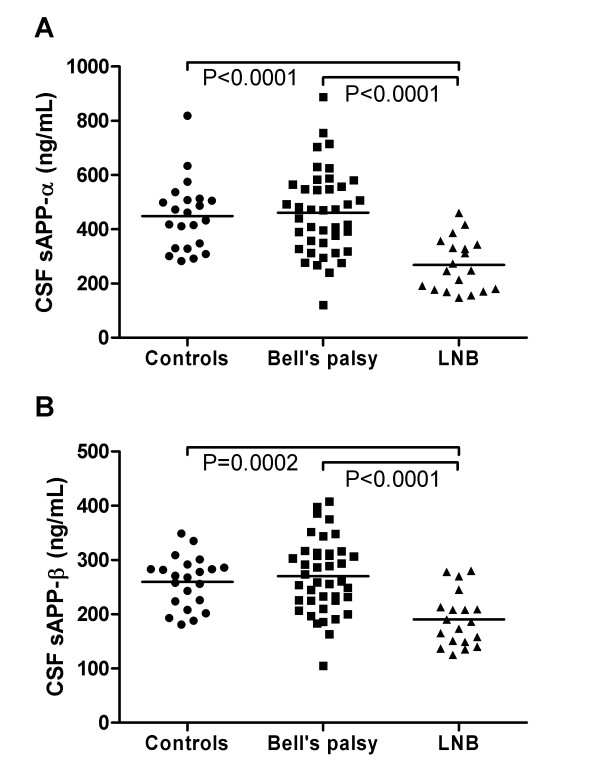
**CSF levels of α-sAPP and β-sAPP in controls, Bell's palsy patients and LNB patients**. Horizontal lines indicate mean values. CSF α-sAPP (panel A) and β-sAPP (panel B) were reduced in LNB patients compared to controls and patients with Bell's palsy.

In the cross-sectional study, LNB patients had lower α-sAPP and β-sAPP than the other groups (Figure 1), but there were no differences in Aβ38, Aβ40 or Aβ42 (Table 2). α-sAPP and β-sAPP correlated to most Aβ peptides in Bell's palsy patients (R = .47-.60, P ≤ .002) and controls (R = .42-.55, P < .05; the only exception was α-sAPP and Aβ38 in controls, where there was a trend towards significance, R = .40, P = .065), but not in LNB patients (P > .05).

**Table 2:** CSF biomarker levels in the cross-sectional study^a^

Group	Aβ38 (ng/L)	Aβ40 (ng/L)	Aβ42 (ng/L)	T-tau (ng/L)	P-tau (ng/L)	**NFL**^**b**^
Controls	779 (323-1767)	6040 (3372-9885)	531 (240-992)	158 (74-553)	36 (20-99)	2/22

LNB	570 (270-2106)	5436 (3456-9465)	434 (198-978)	129 (75-335)	31 (22-71)	10/19^d^

Bell's palsy	694 (108-1615)	5819 (1316-9780)	440 (71-1010)	155 (75-420)	44^c^ (15-80)	6/42^e^

CSF, cerebrospinal fluid; LNB, Lyme neuroborreliosis; ^a^data presented as median (range); ^b^number of patients with CSF concentrations above the detection limit 125 ng/L; ^c^P = .02 vs LNB; ^d^P = .002 vs controls; ^e^P = .002 vs LNB

**Figure 2:**
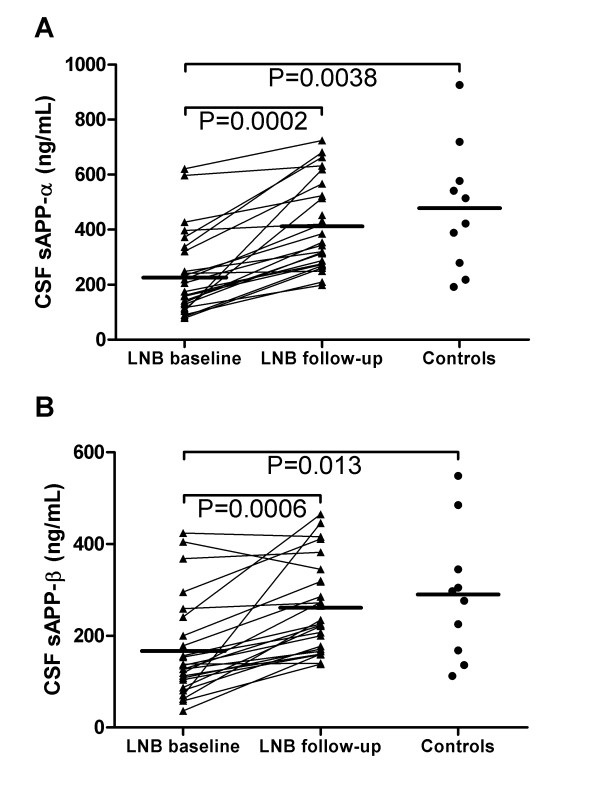
**CSF levels of α-sAPP and β-sAPP in LNB patients with follow-up and controls**. Horizontal lines indicate mean values. CSF α-sAPP (panel A) and β-sAPP (panel B) were reduced in LNB patients compared to controls at baseline, and increased at follow-up.

In the longitudinal study, LNB patients had lower baseline levels of α-sAPP and β-sAPP (Figure 2) and Aβ peptides than controls (Table 3). α-sAPP and β-sAPP increased after treatment (Figure 2), while Aβ levels were unaffected (Table 3). Conversely to what was seen in the cross-sectional study, α-sAPP and β-sAPP correlated to all Aβ peptides in LNB in the longitudinal study (R = .71-.98, P < .001), but not in controls (P > .05).

**Table 3:** CSF biomarker levels in the follow-up study^a^

Group	Aβ38 (ng/L)	Aβ40 (ng/L)	Aβ42 (ng/L)	T-tau (ng/L)	P-tau (ng/L)
Controls	891 (279-1715)	6676 (3450-9672)	672 (293-1193)	349 (171-552)	47 (23-69)

LNB baseline	561^b^ (254-1609)	4840^c^ (2726-8857)	378^d^ (211-997)	229^e^ (171-526)	30^c^ (16-60)

LNB follow-up	510^f^ (234-1562)	4310^g^ (2449-8690)	310^h^ (171-894)	235^i^ (171-532)	33^j,k^ (16-74)

CSF, cerebrospinal fluid; LNB, Lyme neuroborreliosis; ^a^data presented as median (range); ^b^P = .015 vs controls; ^c^P = .006 vs controls; ^d^P = .013 vs controls; ^e^P = .01 vs controls; ^f^P = .021 vs controls; ^g^P = .005 vs controls; ^h^P = .004 vs controls; ^i^P = .031 vs controls; ^j^P = .041 vs controls; ^k^P = .004 vs LNB baseline.

Aβ38 correlated to Aβ40 (R = .91-.97, P < .001) and Aβ42 (R = .74-.92, P < .001), and Aβ40 correlated to Aβ42 (R = .69-.93, P ≤ .001), in all groups. These correlations were expected, since none of the subjects had any known amyloid plaque pathology, which could have entrapped Aβ40 and Aβ42 in the brain parenchyma. In the absence of plaques, these Aβ peptides are likely to diffuse at similar rates to the CSF. α-sAPP and β-sAPP correlated in all groups (R = .88-.95, P < .001), indicating that the pathways producing these peptides are tightly synchronized *in vivo*.

### Total-tau, Phospho-tau and NFL

LNB and Bell's palsy patients had similar T-tau levels in the cross-sectional study, indicating that there was no significant difference in the amount of cortical axonal damage between the groups (Table 2). However, LNB patients had lower P-tau and higher NFL, the latter indicating damage to myelinated axons (Table 2). In the longitudinal study, LNB patients had lower P-tau than controls (Table 3). T-tau and P-tau correlated in all groups (R = .84-.99, P < .001).

### Blood-brain barrier function

In acute disease stage, LNB patients had elevated albumin ratio, reflecting blood-brain barrier dysfunction, which improved after treatment (Table 1). In the cross-sectional study, albumin ratio correlated to β-sAPP in controls (R = .55, P = .018) and to NFL in LNB patients (R = .51, P = .039). In the longitudinal study, albumin ratio correlated inversely to α-sAPP and β-sAPP in LNB patients at baseline (R = -.49, P = .027; R = -.49, P = .028). The correlations seen in LNB could reflect that all these measurements monitor the severity of pathology, rather than a dependency on blood-brain barrier function for sAPP and NFL.

## Discussion

We found signs of altered brain amyloid metabolism in LNB. CSF levels of the soluble APP-fragments α-sAPP and β-sAPP were decreased in the acute stage of the disease, and increased following treatment with antibiotics. Patients with Bell's palsy had no inflammatory activity in CSF and higher CSF α-sAPP and β-sAPP concentrations than LNB patients. These findings add to previous evidence of altered amyloid metabolism in primary inflammatory CNS diseases such as MS, cerebral SLE and HIV with cerebral engagement. In MS, α-sAPP and β-sAPP levels were significantly lower in patients with ongoing or recent disease exacerbation compared to patients in stable remission [3]. In light of these findings, we suggest that CSF levels of α-sAPP and β-sAPP may be generally reduced in diseases with pronounced neuroinflammation. This should be compared to what is seen in AD. Although AD is primarily a neurodegenerative condition, it also has strong inflammatory components [12]. The dense core amyloid plaques in AD brain tissue are surrounded by activated microglia [13]. However, although these cells may phagocytose Aβ it is yet unknown precisely how the inflammatory activity affects amyloid metabolism in AD. Net effects on sAPP proteins differ from what is seen in MS, cerebral SLE and CNS infections, since AD patients have unaltered or even elevated CSF levels of α-sAPP and β-sAPP [2,14,15]. Regarding Bell's palsy, it is an important differential diagnosis to LNB. Although the primary aim of our study was not to assess the diagnostic performance of the amyloid markers, we think it is interesting to note the striking differences between facial palsy caused by LNB and Bell's palsy.

Although low α-sAPP and β-sAPP levels appear to be consistent findings in diseases with pronounced neuroinflammation, the picture becomes more complex when we turn to Aβ peptides. All subjects in the cross-sectional study in the current project had similar Aβ, but in the longitudinal study LNB patients had lower Aβ levels than controls. Note that the LNB patients differed in neurological symptoms between these two parts of the study. Other studies have found decreased Aβ42 in MS, bacterial meningitis and HIV with cerebral engagement [16] but not in viral meningitis [3,17]. Thus, the etiology, duration or severity of the neuroinflammation might affect its influence on Aβ metabolism. In AD, CSF levels of Aβ42 are decreased, which is often explained by accumulation of Aβ42 in plaques [18]. However, low Aβ42 levels may also be seen in Creutzfeld-Jakob's disease, where the abundance of plaques is low [19]. Thus, other mechanisms than plaque accumulation could contribute to low CSF Aβ42 levels in neurodegenerative diseases.

In principle, reduced levels of CSF sAPP could be caused by 1) decreased expression of APP, 2) decreased processing of APP into sAPP, 3) decreased clearance of sAPP into CSF, or 4) increased degradation of sAPP. Presently, it is not possible to draw definite conclusions on which of these mechanisms that operate in neuroinflammation. In cases where no clear reductions in CSF Aβ peptides are seen, such as facial palsy LNB, a decreased APP expression is unlikely. Likewise, altered APP processing with lower β-secretase activity would cause lower levels of both β-sAPP and Aβ peptides. Regarding the third possibility, decreased clearance of sAPP into CSF, it is known that Aβ42 under certain conditions might be trapped in interstitial drainage pathways [20]. It is not known if this is also the case for sAPP. Finally, increased degradation of sAPP in neuroinflammation remains a vital possibility. Little definite data exist on the physiological roles of sAPP, although α-sAPP has been attributed with neuroprotective properties [21]. Particularly interesting in the context of neural damage is a recently described neurotoxic N-terminal APP-fragment, located within the sAPP-sequence, capable of inducing axonal degeneration through interaction with axonal DR6 receptors [22]. Further research should be undertaken to explore whether α-sAPP and β-sAPP share these axonal degenerative properties, or if they are metabolised into DR6-activating peptides in neuroinflammation.

In the follow-up part of the present study LNB patients improved clinically after oral doxycycline, with dramatic effects on the radiculitic pain and a slower recovery of peripheral paresis. CSF analysis with decreased CSF inflammation measured as number of monocytic cells and normalization of the blood-brain barrier measured as albumin ratio support the excellent therapeutic effect. The LNB patients were younger than the controls, which might affect the statistical analysis of some parameters. However, in the study on MS referred to earlier, CSF levels of α-sAPP and β-sAPP decreased in both patients and controls during a 10 year follow-up [3]. Thus, it is unlikely that the age difference explains the low baseline levels of sAPP in young LNB patients compared to older controls.

Regarding markers of neural damage and tau pathology, we confirm earlier findings of increased CSF NFL in some LNB patients, which indicates acute damage to large myelinated axons [23]. White matter lesions have also been described previously in LNB [24]. However, the CSF NFL levels were low compared to other CNS diseases such as herpes encephalitis [25]. NFL correlated to albumin ratio, likely since both axons and the blood-brain barrier are more affected in severe cases. For CSF T-tau, considered a marker of damage to cortical axons [26], we found no difference between LNB and Bell's palsy. This suggests that the neural damage in LNB is not located to cortical structures. T-tau levels were even lower in LNB patients than in controls. Since CSF T-tau increases with age, this could partly be explained by the age-difference between LNB patients and controls [27]. CSF P-tau was decreased in LNB patients, and increased after treatment in the follow-up study. Thus, the acute neuroinflammation may have affected tau phosphorylation.

## Conclusions

We report an association of cerebral amyloid metabolism with LNB, with decreased CSF levels of soluble APP-fragments in the acute disease stage. Besides possible implications for diagnosis of LNB, these findings strengthen the case for a general involvement of amyloid in neuroinflammation and suggest that a physiological role of APP could be to participate in inflammatory pathways. We also confirm previous findings of elevated CSF NFL in LNB, suggesting damage to myelinated axons. Further research on amyloid metabolism in neuroinflammation will likely shed more light on the functions of APP in the adult human nervous system.

## Competing interests

KB has on one occasion received a consulting fee for an advisory board meeting from Innogenetics. The other authors have no conflicts of interest.

## Authors' contributions

NM, DB, LH, KB and HZ had the idea for this particular study and participated in the conception and design of the study. NM and DB drafted the paper. LH and DB recruited patients and analyzed the clinical effect. HA, KB, RA and HZ made important revisions to the manuscript. All authors read and approved the final manuscript.

## Pre-publication history

The pre-publication history for this paper can be accessed here:

http://www.biomedcentral.com/1471-2377/10/51/prepub
